# A Bayesian view of murine seminal cytokine networks

**DOI:** 10.1371/journal.pone.0188897

**Published:** 2017-11-30

**Authors:** Michelle L. Johnson, Tathagata Dasgupta, Nadia Gopichandran, Sarah L. Field, Nicolas M. Orsi

**Affiliations:** 1 Department of Pathology and Tumour Biology, Leeds Institute of Cancer and Pathology, The University of Leeds, Leeds, United Kingdom; 2 Sleker, Brookline, Massachusetts, United States of America; 3 Department of Systems Biology, Harvard Medical School, Boston, Massachusetts, United States of America; 4 Ostara Biomedical Ltd, Liverpool, United Kingdom; University of California, San Diego, UNITED STATES

## Abstract

It has long been established that active agents in seminal fluid are key to initiating and coordinating mating-induced immunomodulation. This is in part governed by the actions of a network of cytokine interactions which, to date, remain largely undefined, and whose interspecific evolutionary conservation is unknown. This study applied Bayesian methods to illustrate the interrelationships between seminal profiles of interleukin (IL)-1alpha, IL-1beta, IL-2, IL-4, IL-5, IL-6, IL-9, IL-10, IL-12 (p70), IL-13, IL-17, eotaxin, granulocyte-colony stimulating factor (G-CSF), granulocyte macrophage-colony stimulating factor (GM-CSF), interferon (IFN)-gamma, keratinocyte-derived chemokine (KC), monocyte chemoattractant protein (MCP-1), macrophage inflammatory protein (MIP-1) alpha, MIP-1beta, regulated on activation normal T cell expressed and secreted (RANTES), tumour necrosis factor (TNF)-alpha, leptin, inducible protein (IP)-10 and vascular endothelial growth factor (VEGF) in a rat model. IL-2, IL-9, IL-12 (p70), IL-13, IL-18, eotaxin, IFN-gamma, IP-10, KC, leptin, MCP-1, MIP-1alpha and TNF-alpha were significantly higher in serum, whilst IL-1beta, IL-5, IL-6, IL-10, IL-17, G-CSF and GM-CSF were significantly higher in seminal fluid. When compared to mouse profiles, only G-CSF was present at significantly higher levels in the seminal fluid in both species. Bayesian modelling highlighted key shared features across mouse and rat networks, namely TNF-alpha as the terminal node in both serum and seminal plasma, and MCP-1 as a central coordinator of seminal cytokine networks through the intermediary of KC and RANTES. These findings reveal a marked interspecific conservation of seminal cytokine networks.

## Introduction

It is well established that seminal plasma governs the development of maternal reproductive tract immunomodulation essential for the establishment of pregnancy and maternal tolerance of the foetal allograft [1–5]. This process is driven by immunomodulatory moieties such as cytokines, steroid binding proteins and prostaglandins, which results in the relocation of immune effector cells to implantation sites and other mucosal surfaces [6–12]. Such changes are thought to inhibit genital tract immune defences, resulting in reduced cell-mediated responses and immunosurveillance [13]. While the primary site of responsiveness to seminal fluid is believed to be the ectocervix in women, [14] maternal responses to semen exposure have been best characterised in murine models, where coitus stimulates a classic inflammatory cascade-like uterine response [11]. This results in chronologically coordinated endometrial epithelial and stromal responses geared to support implantation [7], coordinate immune effector cell recruitment to the luminal epithelium/decidua, and enable the establishment of pregnancy, both by minimising cell-mediated immunity and modulating abortifacient interferon (IFN)-gamma production [15–17]. In mice, coitus has also been shown to induce systemic changes in cytokine profiles, including decreases in serum IFN-gamma and interleukin (IL)-12 (p70), as well as increases in keratinocyte-derived chemokine (KC) and granulocyte-colony stimulating factor (G-CSF) [18].

Despite the key role played by these agents, experimental and observational endeavours have remained largely on individual cytokines. However, cytokines are recognised as operating as networks, exhibiting synergy, antagonism and functional redundancy such that their functional effects must be considered in the context of their putative interactions with other mediators in governing their own concentrations. This degree of control is crucial in ensuring an appropriate post-coital response, yet their functional interactions remain ill-defined. By way of example, perturbations in seminal plasma IL-1beta, IL-4, IL-8, IL-10 and IFN-gamma profiles have been correlated with infertility in men, yet their interrelationships remain to be described [19, 20]. There is a paucity of data relating to the extent to which these seminal cytokines profiles are conserved across species, if at all. Although mouse and rat cytokines display greater biological cross-reactivity and functional homology than evolutionarily distant species [21], little is known about the potential functional interrelationships between these mediators in the *in vivo* setting. This study therefore aimed (i) to characterise physiological cytokine profiles in rat seminal fluid and to compare it to that previously determined in mice and (ii) to apply Bayesian modelling approaches to establish possible hierarchical/functional interrelationships across cytokines in both species.

## Materials and methods

### Sample collection

This study was carried out in strict accordance with the Animals (Scientific Procedures) Act, 1986 (ASPA) and was approved by The University of Leeds. Sexually mature male Wistar rats (body mass >350g; *n* = 20) were sourced from Charles River (UK). These were then housed individually with ad libitum access to water and Rodent Diet (BK001 (E) 801960, Special Diets Service Essex, UK). The lighting cycle was 14 h:10 h light:dark, and humidity and temperature were maintained at 55–65% and 21.5 ± 1°C. These were sacrificed by exposure to a rising concentration of carbon dioxide under Schedule 1 of the ASPA. Seminal fluid was collected from isolated seminal glands, weighed (to correct for subsequent dilution), then immediately diluted with 200 μl sterile PBS with 0.5% BSA and vortexed for 45 seconds [15]. In parallel, serum was isolated from blood collected by post mortem cardiac puncture which was allowed to clot on ice. All samples were centrifuged at 9,000 rpm (5,600 g) for 3 min using a microfuge (Micro Centaur, MSE Scientific, Loughborough, UK), and supernatants stored at -80°C until analysis.

### Multiplex cytokine analysis

Rat seminal fluid and serum samples were profiled for IL-1alpha, IL-1beta, IL-2, IL-4, IL-5, IL-6, IL-9, IL-10, IL-12 (p70), IL-13, IL-17, eotaxin, G-CSF, granulocyte macrophage-colony stimulating factor (GM-CSF), IFN-gamma inducible protein (IP)-10, KC, leptin, monocyte chemoattractant protein (MCP)-1, macrophage inflammatory protein (MIP)-1alpha, MIP-1beta, regulated on activation normal T cell expressed and secreted (RANTES), tumour necrosis factor (TNF)-alpha and vascular endothelial growth factor (VEGF) by 24-plex fluid-phase immunoassay (LincoPlex, Millipore, Livingston, UK) run on a Luminex-100 cytometer (Luminex Corporation, Austin, TX, USA), equipped with Bio-Plex software (BioRad Laboratories, Hemel Hempstead, UK). Serum diluent dilution was adjusted to 1:1 in order to maximise sensitivity to baseline levels [18]. Multiplex analysis of mouse seminal plasma was performed as part of a previous study [15] and data used with permission.

### Data presentation and statistical analysis

Cytokine levels were corrected for dilution as previously described for seminal plasma [20]; all were expressed in pg/ml as mean ± SEM. Data distributions were assessed by Shapiro-Wilk tests and significant differences between groups were determined using paired samples *t*-tests or related-samples Wilcoxon signed-rank tests, as appropriate, with correction for multiple comparisons applied using Holm’s sequential method (SPSS, IBM Corporation, New York, USA).

### Bayesian network construction

Bayesian networks were constructed as previously described in detail [22]; additional information is available (S1 File, S1 Fig). Briefly, seeded, species-specific prior networks were generated in *MetaCore* (GeneGo, http://thomsonreuters.com/en/products-services/pharma-life-sciences/pharmaceuti-calresearch/metacore.html) and combined with text mining results (*Predictionet*; http://www.bioconductor.org/packages/devel/bioc/html/predictionet.html). Structural feedback loops were removed and, prior to performing the Bayesian network analysis, z-score normalizations were applied to the raw data in *Matlab*. High-confidence networks were derived from both prior and experimental data using a machine learning algorithm (*MeV* in Weka; http://www.cs.waikato.ac.nz/ml/weka/). Cytokine profiles were discretised into categorical data and allocated arbitrarily to three mutually exclusive equal frequency, relative concentration bins (low, intermediate, high). The resultant nodes were colour-coded based on the underlying conditional probability tables (see Results). Stringent non-parametric bootstrapping (100 operations) was applied to avoid over- fitting by re-sampling with replacement to estimate network feature confidence. The Bayes (BDe) score was optimised using the Tabu Search algorithm in *Weka* (http://weka.sourceforge.net/manuals/weka.bn.pdf) and the resultant directed acyclic graph visualised using Cytoscape (http://www.cytoscape.org). This approach was applied to both the current rat data and to mouse data from a previous study for the purpose of interspecific comparisons [20]. In order to allow fair comparisons to be made in the latter case, new networks were created using only cytokines measured in both species so as to highlight conserved interrelationships; IP-10, leptin and VEGF were removed from the rat networks, and IL-3 plus IL12 (p40) were removed from the mouse networks. For a more detailed explanation, please see S1 File.

## Results

### Seminal fluid and serum cytokine profiles

The cytokines detected at the highest level in seminal fluid were KC and RANTES, albeit at lower concentrations than in serum, while IL-1alpha, IFN-gamma, IP-10, G-CSF, MIP-1alpha and TNF-alpha were present at very low levels and VEGF was undetectable. The most abundant cytokines in serum were leptin and RANTES, whilst the lowest levels were detected for IL-1alpha, G-CSF, MIP-1alpha and TNF-alpha. Serum IL-6, IL-10 and GM-CSF were undetectable. Many rat seminal fluid cytokine levels were significantly lower than their serum counterparts, including IL-9, IL-13, IL-18, IFN-gamma, KC, MCP-1, MIP-1alpha, TNF-alpha, leptin, IP-10 (*P*<0.001), IL-12 (p70), eotaxin (*P*<0.01) and IL-2 (*P*<0.05). By contrast, seminal fluid levels of IL-5, IL-6, IL-10, IL-17, G-CSF, GM-CSF (*P*<0.001) and IL-1beta (*P*<0.01) were higher than in serum. These data were compared with those of the mouse (Table 1) from a previous study [15]. Across both species, only G-CSF was consistently present at significantly higher levels in seminal fluid (mice, *P*<0.01; rats, (*P*<0.001).

**Table 1:** A comparison of mouse and rat cytokine concentrations in seminal fluid and serum in mice and rats.

	Cytokine concentration in seminal fluid (pg/ml)		Cytokine concentration in serum (pg/ml)	
Cytokine	Mouse	Rat	Mouse	Rat
IL-1alpha	**8.19 ± 1.96**	3.09 ± 1.01	**62.24 ± 0.76**	18.92 ± 6.74
IL-1beta	**87.48 ± 9.04**	20.14 ± 0.84	**113.76 ± 4.25**	6.84 ± 2.47
IL-2	3.03 ± 0.49	**27.74 ± 3.29**	9.41 ± 1.47	**234.45 ± 90.66**
IL-3	0.35 ± 0.04	*	0.00	*
IL-4	0.11 ± 0.01	**19.63 ± 1.04**	0.10 ± 0.03	**20.00 ± 2.54**
IL-5	0.56 ± 0.07	**9.03 ± 0.82**	**4.82 ± 0.62**	0.25 ± 0.17
IL-6	3.63 ± 0.44	**147.01 ± 7.73**	**10.15 ± 2.76**	0.00
IL-9	**135.14 ± 33.47**	53.36 ± 4.35	285.95 ± 27.44	**387.39 ± 23.88**
IL-10	19.95 ± 3.36	**111.39 ± 6.68**	**16.03 ± 4.02**	0.00
IL-12 (p40)	5.25 ± 0.53	*	309.65 ± 30.19	*
IL-12 (p70)	10.91 ± 1.08	**52.98 ± 3.94**	76.91 ± 10.84	**108.24 ± 10.18**
IL-13	**20.64 ± 1.86**	7.70 ± 1.28	171.69 ± 16.50	**308.64 ± 21.63**
IL-17	5.10 ± 0.90	**14.99 ± 0.80**	**161.31 ± 26.88**	1.25 ± 0.64
IL-18	*	6.17 ± 0.79	*	113.73 ± 26.31
Eotaxin	**857.22 ± 73.85**	34.04 ± 1.35	**401.33 ± 38.07**	47.50 ± 3.37
G-CSF	**45.03 ± 3.33**	1.47 ± 0.06	**30.32 ± 3.25**	0.09 ± 0.09
GM-CSF	4.16 ± 0.39	**39.69 ± 2.04**	**30.79 ± 2.39**	0.00
IFN-gamma	**46.38 ± 3.95**	2.80 ± 0.39	**388.72 ± 29.30**	46.91 ± 5.82
KC	37.17 ± 3.56	**229.24 ± 24.48**	26.70 ± 1.27	**643.20 ± 25.04**
MCP-1	30.23 ± 2.65	**60.73 ± 2.16**	305.54 ± 25.43	**380.75 ± 20.32**
MIP-1alpha	**114.32 ± 8.31**	0.12 ± 0.02	**108.21 ± 11.94**	14.97 ± 1.13
MIP-1beta	6.68 ± 1.36	*	14.44 ± 3.23	*
RANTES	**618.62 ± 84.17**	285.05 ± 22.53	0.58 ± 0.58	**4398.42 ± 1831.59**
TNF-alpha	**102.27 ± 9.11**	2.21 ± 0.16	**224.84 ± 27.02**	11.94 ± 0.93
Leptin	*	41.96 ± 2.07	*	9498.56 ± 791.89
IP-10	*	4.03 ± 0.28	*	27.31 ± 2.54
VEGF	*	0.00	*	2.04 ± 1.36

Numbers in bold indicate significantly higher concentrations. (* not measured) [data for mice provided for comparison from 15].

### Bayesian networks

**Fig 1:**
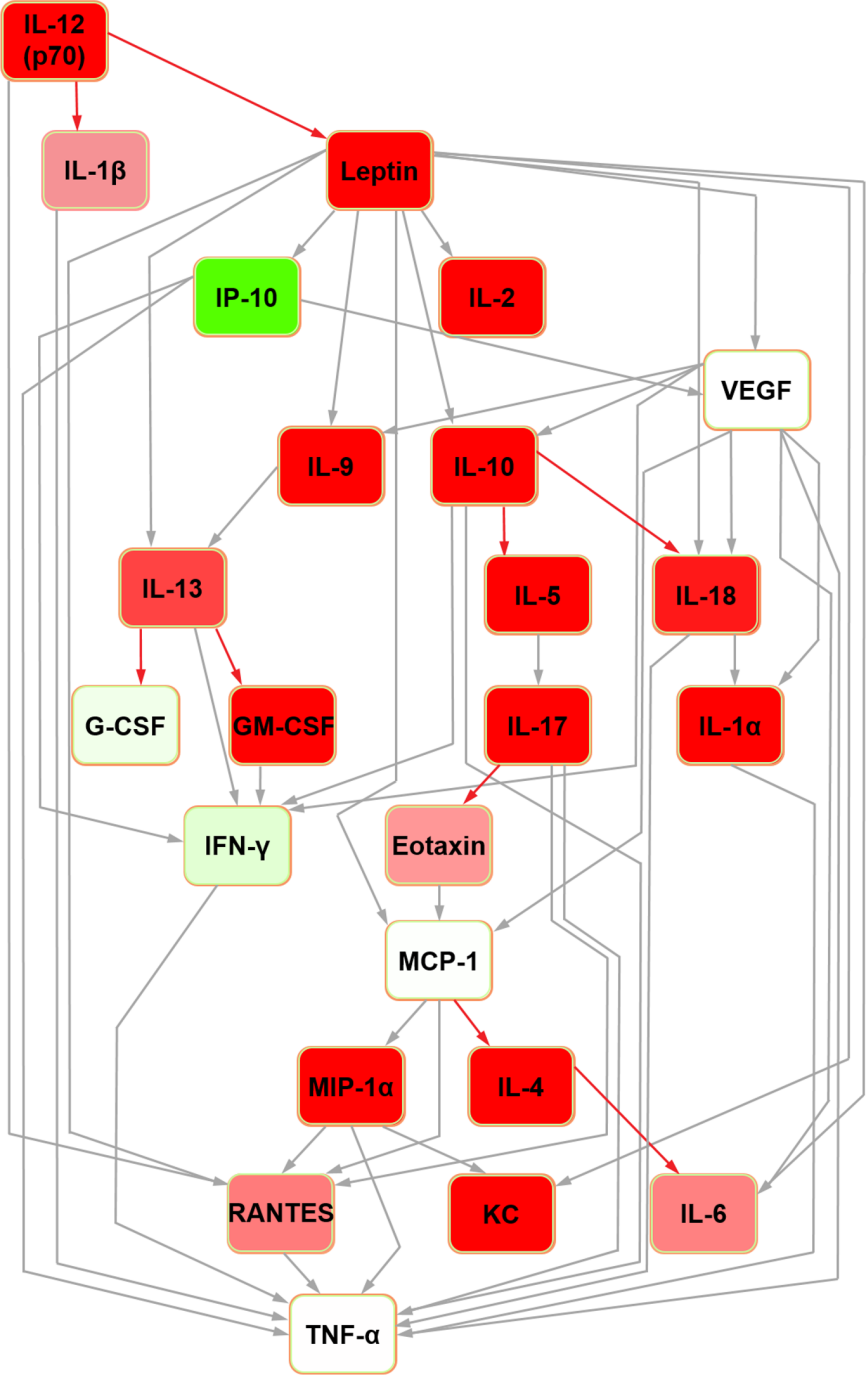
Bayesian network depicting cytokine interrelationships in rat seminal fluid. The nodes are colour-coded according to the conditional probability of corresponding mediator relative concentrations being high (green), low (red) or medium (white) concentration given the state(s) of their parent nodes. Relative to the white colour, the normalised concentration (low or high) determines the intensity of the node colour. Grey-coloured confidence level edges (causal connecting arrows between nodes) represent a confidence level of 80%; red edges are below this level, based upon the confidence analysis of the Bayesian result.

**Fig 2:**
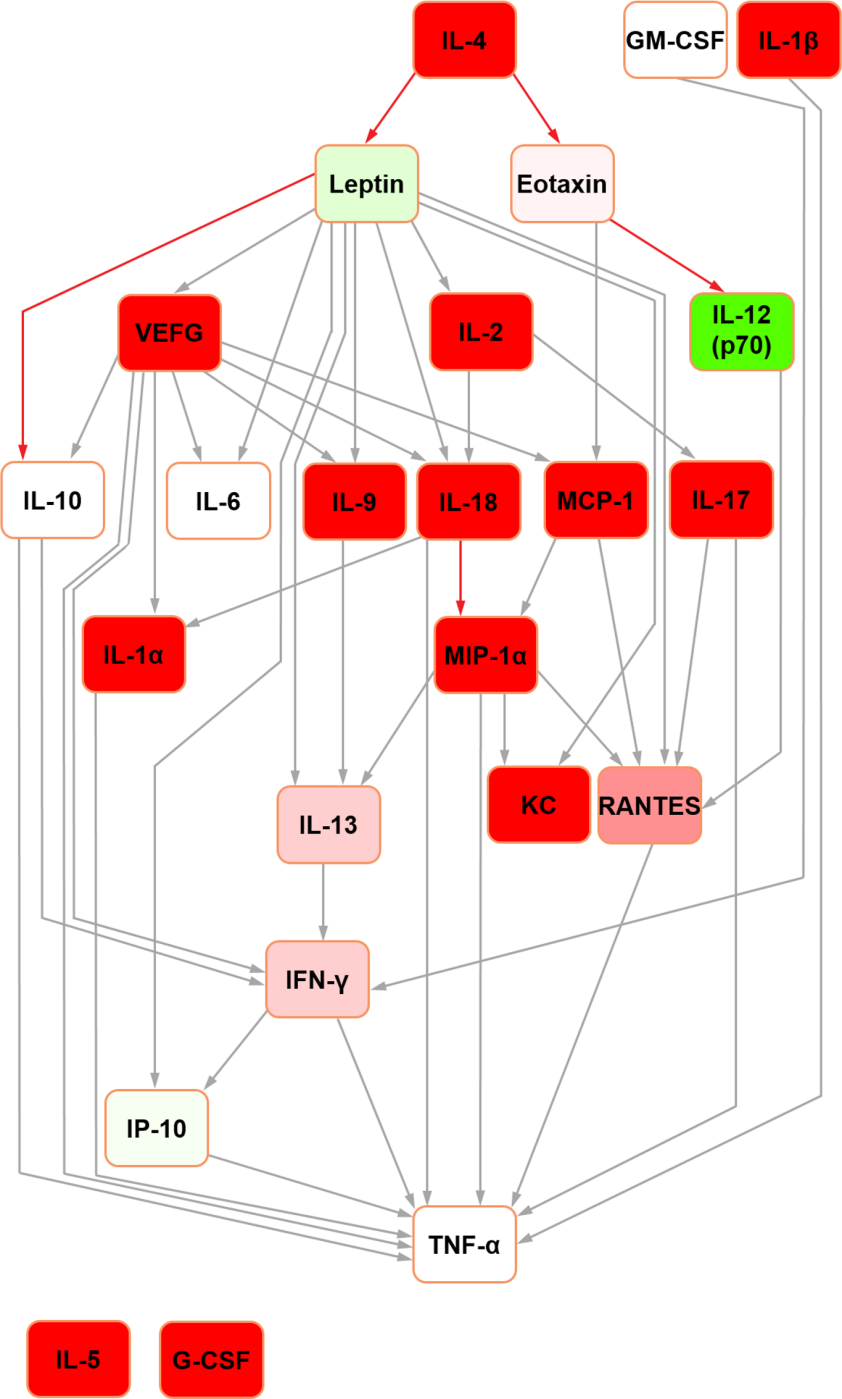
Bayesian network depicting cytokine interrelationships in rat serum. (See Fig 1 legend for details regarding colour-coding).

For the sake of clarity, detailed definitions of the nature of Bayesian network structure and a glossary of terms can be found in S1 File. In the rat seminal fluid cytokine network (Fig 1), IL-12 (p70) was the parent node and TNF-alpha was the terminal node. It contained four nodes with hub/hub-like features (hereafter collectively referred to as ‘hub’ for simplicity; the reader is referred to [22] for detailed definitions): IL-10, IL-13, VEGF and MCP-1. In the rat serum Bayesian network (Fig 2), IL-5 and G-CSF were orphan nodes (i.e. not connected to the rest of the network). IL-4 was the parent node, with edges connecting to leptin and eotaxin. The network assembled around five hubs: IL-10, IL-18, IFN-gamma, MCP-1 and MIP-1alpha with all but MCP-1 feeding into the terminal node (TNF-alpha) directly.

**Fig 3:**
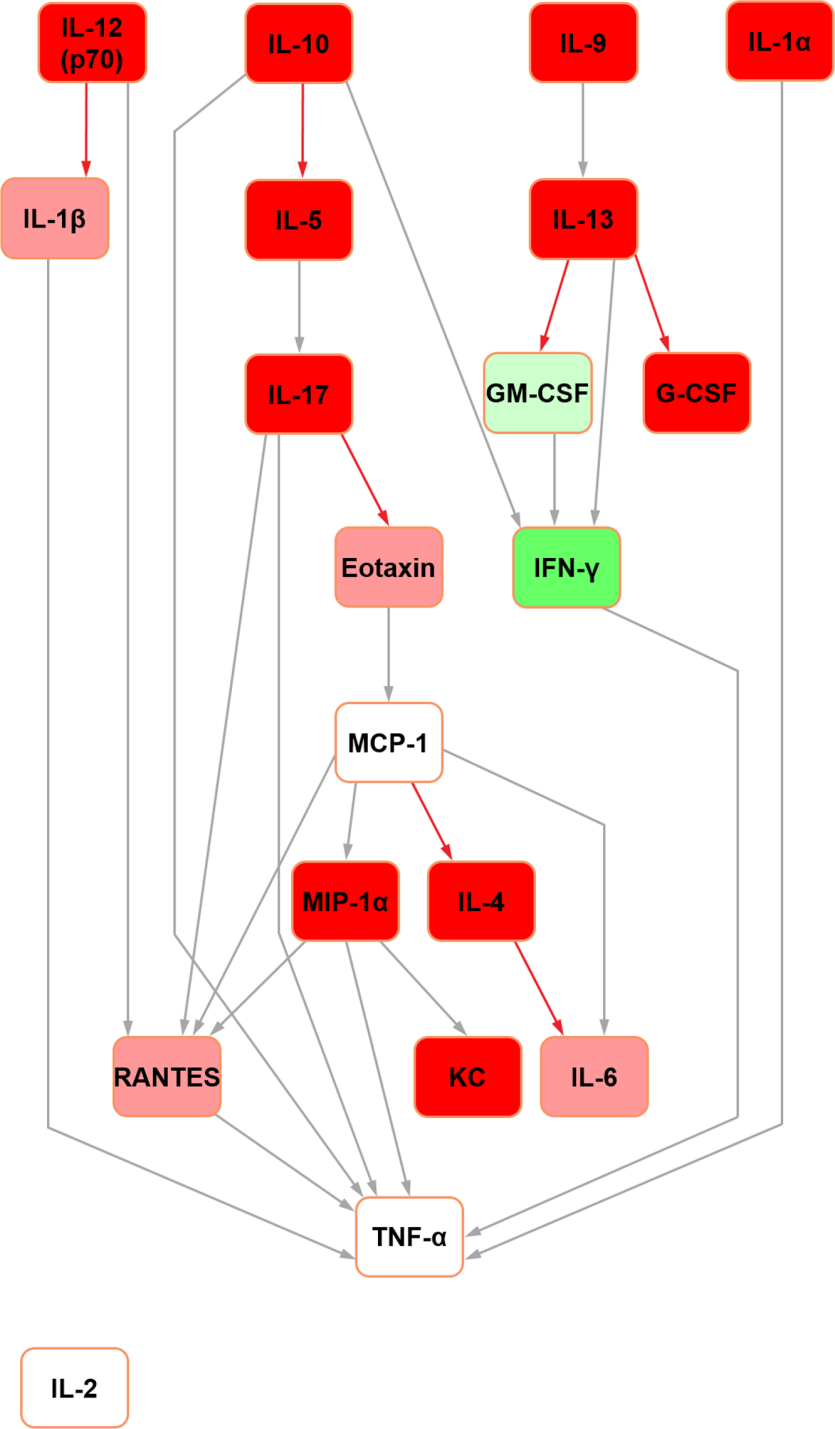
Bayesian network depicting cytokine interrelationships in rat seminal fluid, with nodes not common with the mouse network removed. (See Fig 1 legend for details regarding colour-coding).

**Fig 4:**
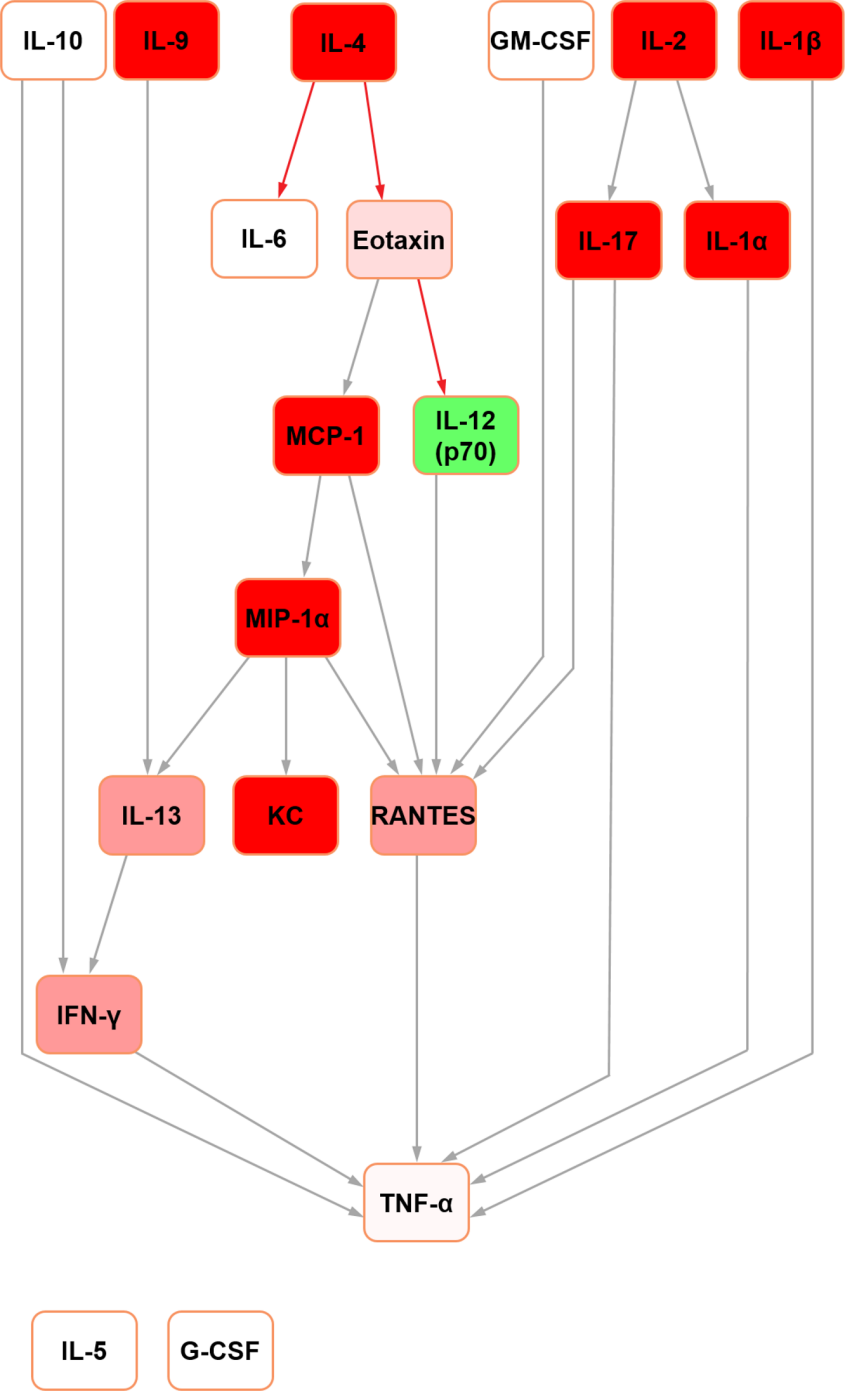
Bayesian network depicting cytokine interrelationships in rat serum, with nodes not common with the mouse network removed. (See Fig 1 legend for details regarding colour-coding).

Further Bayesian networks were constructed for subsequent comparison with their mouse counterparts [15] by excluding cytokines which were not measured in both species due to availability of analytical platform targets (i.e. IL-3, IL-12 (p40) and MIP-1beta in the rat; IL-18, IP-10, leptin and VEGF in the mouse) (Figs 3 and 4). In the present analysis, in both seminal fluid and serum, TNF-alpha remained the terminal node. In rat seminal fluid, the removal of leptin from the modelling caused some restructuring: IL-2 became orphaned, and there were no hub nodes (although RANTES and IFN-gamma both had multiple inputs). Despite these changes, multiple shared structural features were retained across the two seminal fluid networks (Figs 1 and 3), which were particularly evident downstream of IL-10. Rat serum networks also demonstrated a high level of conservation between the restructured networks after leptin removal (Figs 2 and 4); the same nodes were orphaned (IL-5 and G-CSF) and TNF-alpha remained as the terminal node.

**Fig 5:**
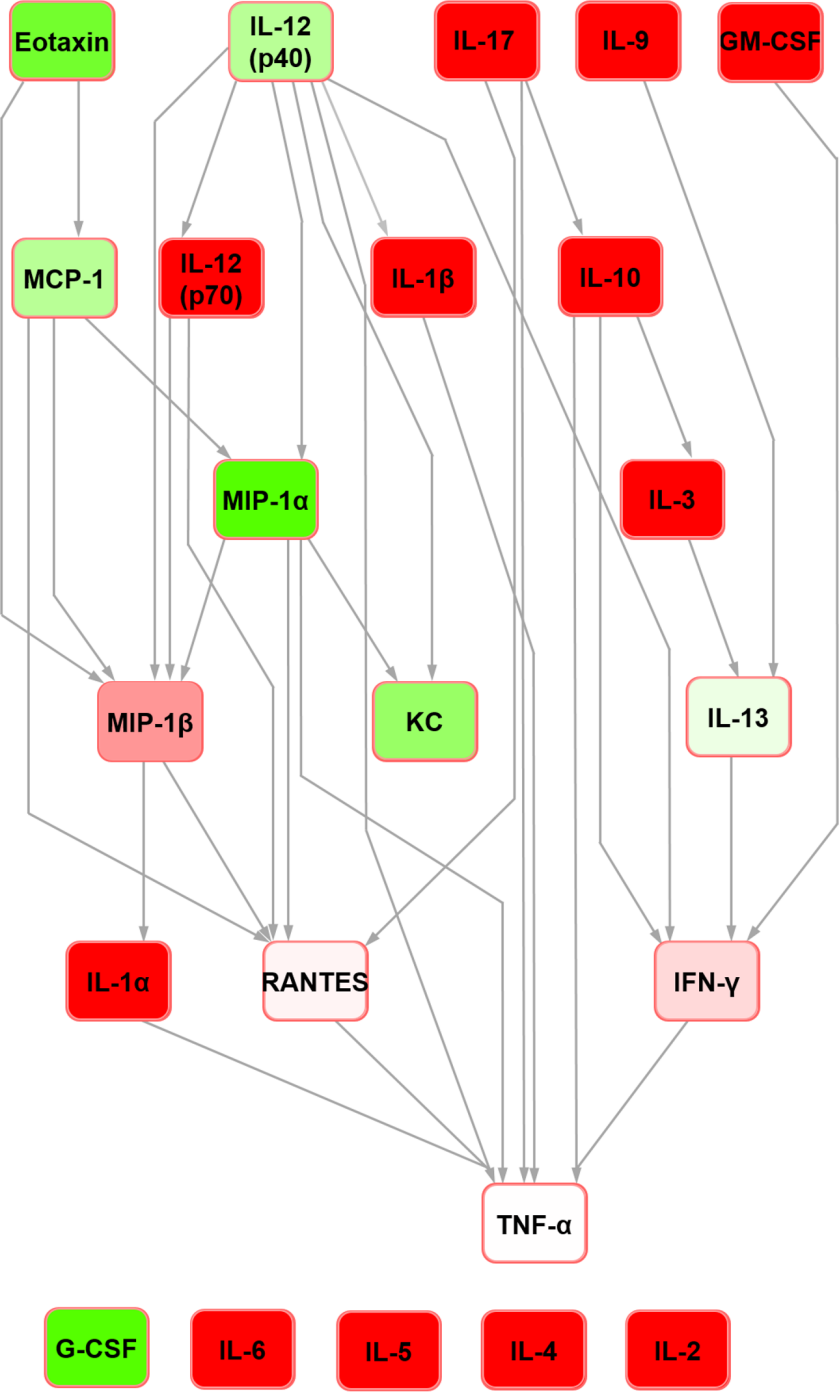
Bayesian network depicting cytokine interrelationships in mouse seminal fluid. (See Fig 1 legend for details regarding colour-coding).

**Fig 6:**
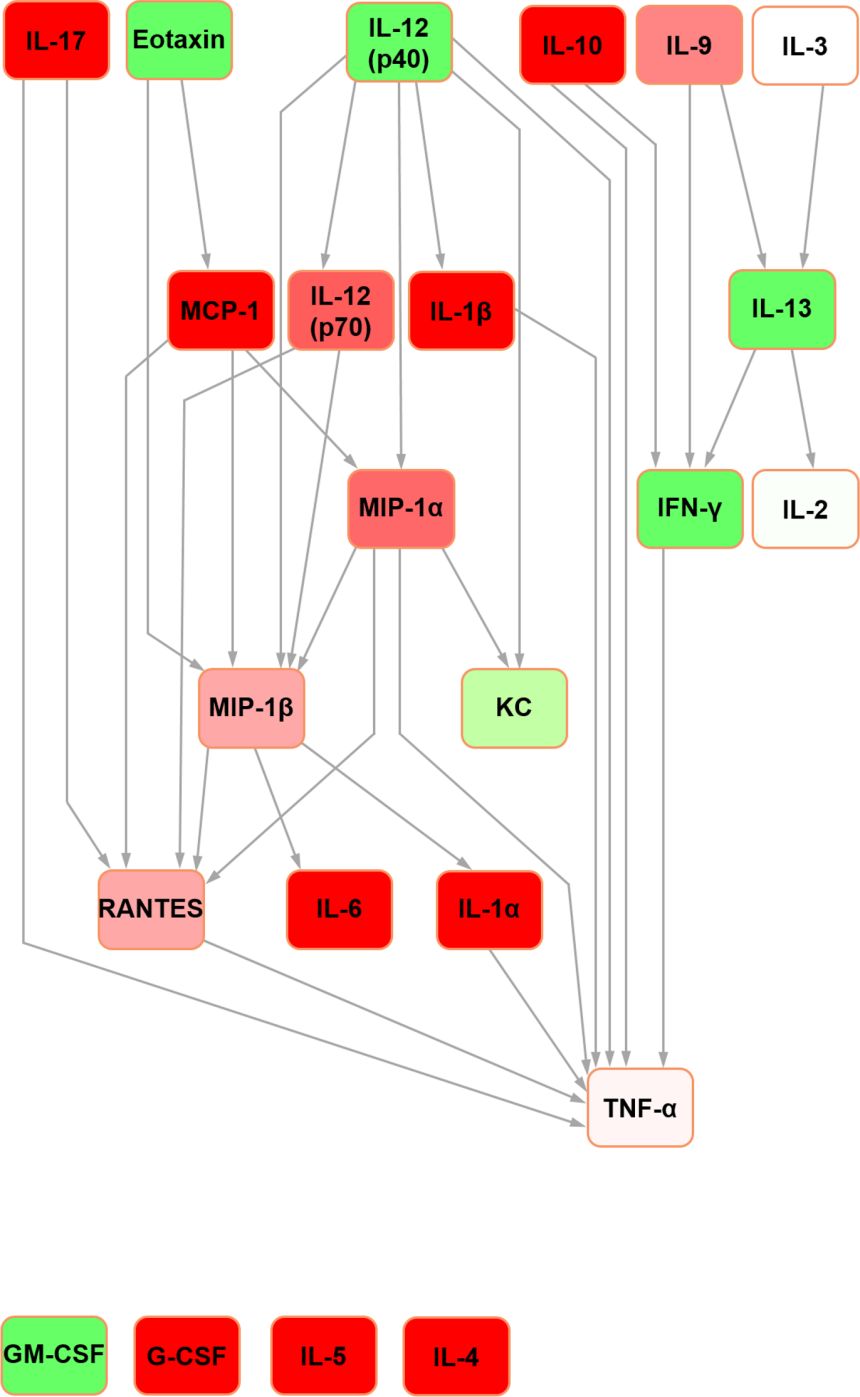
Bayesian network depicting cytokine interrelationships in mouse serum. (See Fig 1 legend for details regarding colour-coding).

The Bayesian network constructed for mouse seminal fluid (raw data used with permission [15]; Fig 5) assembled around two hubs: MIP-1alpha and MIP-1beta. G-CSF, IL-2, IL-4, IL-5 and IL-6 were orphaned from the network. In mouse serum (Fig 6), three hubs were evident: IL-13, MIP-1alpha and MIP-1beta. Both mouse networks featured TNF-alpha as the terminal node. This was a shared feature across both mice and rats, together with the same parent nodes feeding into it. However, while IL-4 was the principal original parent node in the rat serum network, this was orphaned in its mouse counterpart.

**Fig 7:**
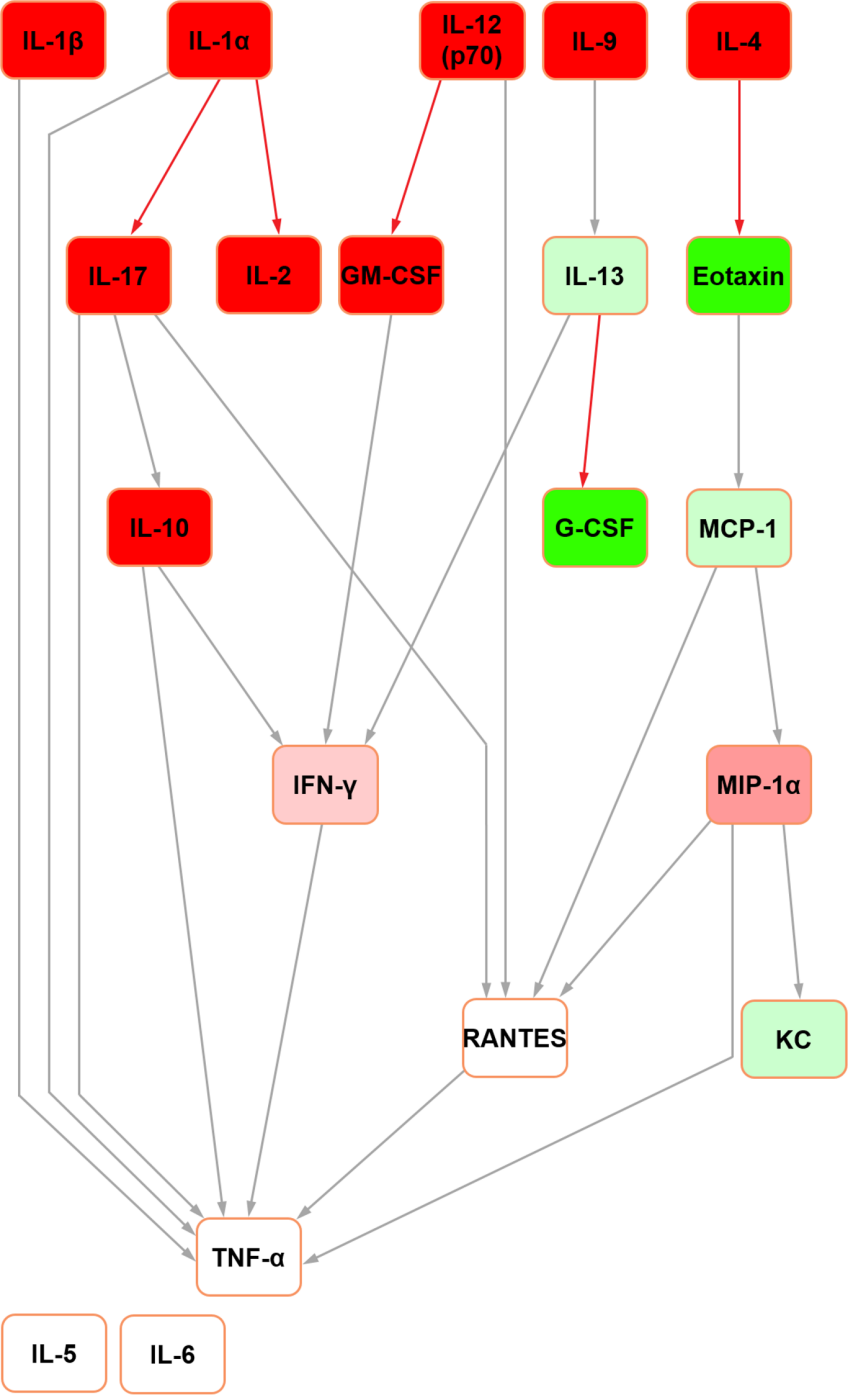
Bayesian network depicting cytokine interrelationships in mouse seminal fluid, with nodes not common with the rat network removed. (See Fig 1 legend for details regarding colour-coding).

**Fig 8:**
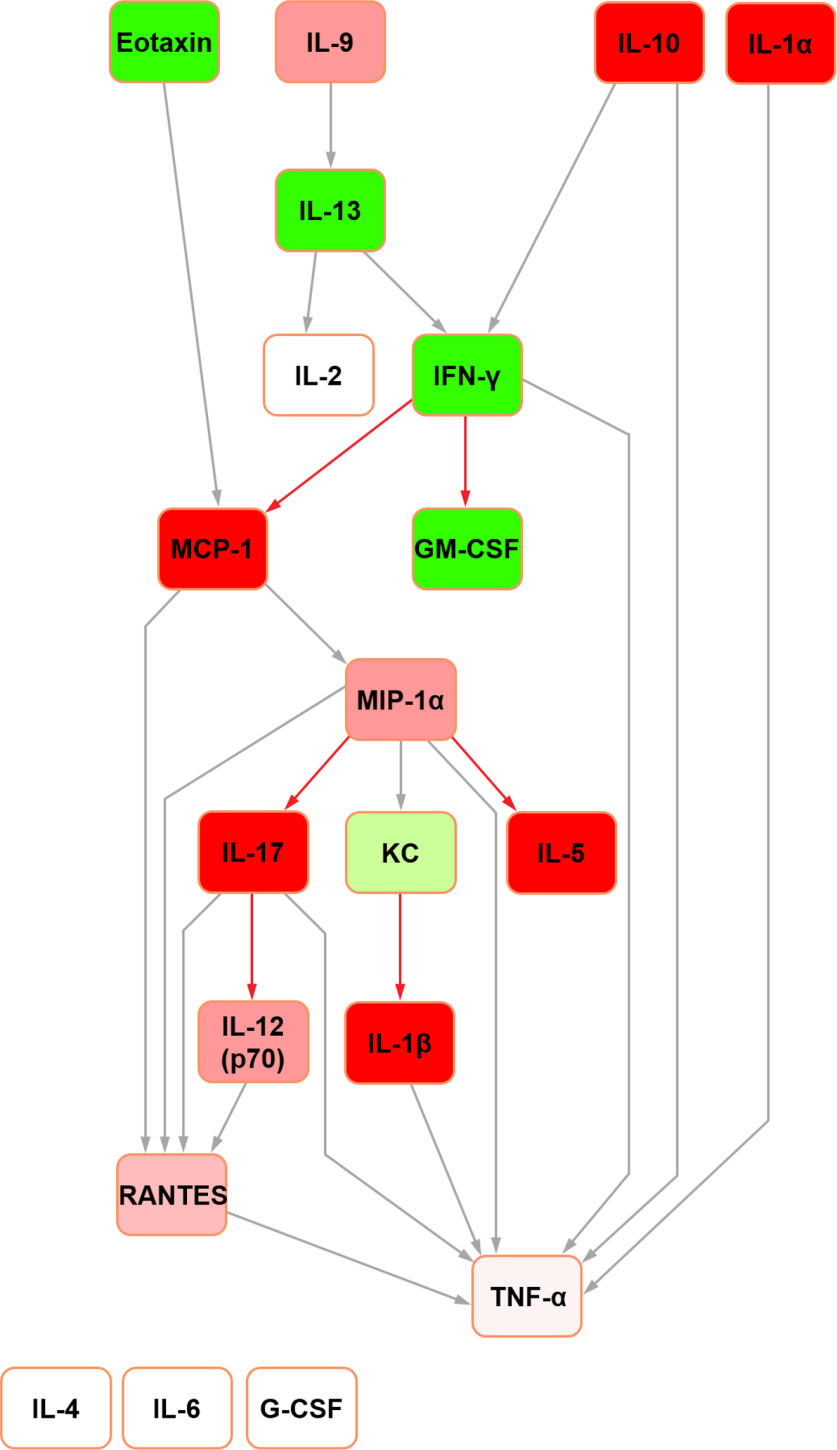
Bayesian network depicting cytokine interrelationships in mouse seminal fluid, with nodes not common with the rat network removed. (See Fig 1 legend for details regarding colour-coding).

In the abridged mouse seminal fluid network (i.e. formulated using mediators profiled for both species to enable a fair interspecific comparison by removing the potential bias of mediators not represented in both systems; see above), IL-5 remained orphaned (Fig 7). Moreover, the removal of MIP-1beta orphaned IL-6 in both seminal fluid (Fig 7) and serum (Fig 8) in the mouse. In the original mouse seminal fluid network (Fig 5), G-CSF was orphaned from the rest of the network; in the abridged network (Fig 7), IL-13 had a directed edge towards G-CSF, which was also seen in the rat (Figs 1 and 3). Shared features across all networks (complete and abridged) and species all included TNF-alpha as the terminal node and MCP-1 edges to MIP-1 alpha and RANTES.

## Discussion

The main findings of this study included: (i) RANTES and KC were the most abundant cytokines in rat seminal fluid; (ii) MCP-1 may be a key regulator of both RANTES and KC in seminal fluid and serum; (iii) high IL-6 and IL-10 levels occur in rat seminal fluid; (iv) G-CSF was the only cytokine found to be present at significantly higher concentrations in both rat and mouse seminal fluid; and (v) TNF-alpha consistently featured as the terminal node to each network. To the best of our knowledge, this is the first time that Bayesian modelling methods have been used to capture the interactions between seminal mediators in order to draw functional inferences about interspecific conserved relationships. In many species, including rodents and humans, the receptivity of the maternal reproductive tract to the conceptus is influenced by factors in seminal plasma that activate lymphocytes, for which the relocation of antigen-presenting cells to the uterus is necessary [23, 24].

As outlined, two of the most abundant seminal fluid and serum cytokines in rats were RANTES and KC. In mice [15] and humans [25], the seminal fluid RANTES concentrations were similarly high. Low levels of RANTES in men have been associated with subfertility relating to the presence of seminal anti-sperm antibodies [26]. By contrast, infertile men without seminal anti-sperm antibodies have RANTES levels similar to their fertile counterparts, pointing to an inappropriate RANTES-related immune response occurring in the genital tract of immunoinfertile men. Both RANTES and its receptors are present in the reproductive tract of women, suggesting that there may be coordination between male and female secreted RANTES in the immunomodulation of early pregnancy [27].

The relatively high rat seminal fluid KC levels may reflect its potential role in post-coital neutrophil chemotaxis and activation. In this respect, female rats mated with seminal vesicle-deficient stud males reportedly have a complete absence of neutrophils in the uterine luminal cavity [28]. Taken together, it may be reasonable to assume that high levels of RANTES and KC may thus orchestrate neutrophil relocation to the site of semen deposition and, in line with these findings, the rat Bayesian networks suggest that MCP-1 may be a key regulator of both RANTES (both directly and indirectly) and KC (indirectly) levels in seminal fluid and serum.

MCP-1 has been proposed to influence RANTES levels during lactation [22] as well as regulating KC production during post-injury inflammatory responses in mice [29]. More specifically, MCP-1 has been proposed to lead to increases in both circulatory RANTES and KC levels [22]. In both rat networks, chemotactic MCP-1 was a hub node and possible key signal integrator. MCP-1 levels were relatively high in both rat and mouse sera and, like RANTES, MCP-1 expression has been shown to be high in the mouse uterus on day 1 *post coitum*, making it a candidate of the female post-mating response leading to macrophage recruitment [30]. In this regard, human cervical cells have been documented to respond to seminal plasma with an increase in MCP-1 at both the transcriptional and protein level [14]. Together, these studies suggest a key regulatory role for MCP-1 in seminal networks in both rodents and humans, and that exposure of the female reproductive tract to seminal plasma may elicit further increases in local MCP-1 and, in turn, uterine RANTES and KC levels.

While previous reports have indicated that seminal eotaxin levels are high in both mice [15] and humans [25], rat profiles are comparatively lower. Eotaxin purportedly acts in conjunction with IL-1beta, IL-9 and MIP-1alpha to initiate endometrial stromal cytokine synthesis, including IL-6 and GM-CSF which, in turn, recruit and activate antigen-presenting cells to process paternal ejaculate antigens [31, 32]. The comparatively low levels of the former mediators in rat seminal fluid may be offset by the higher levels of ‘downstream’ IL-6 and IL-10. In mice, although IL-6 is present at low concentrations in seminal fluid, interactions with endometrial epithelial cells induces its production as well as that of GM-CSF, KC and MCP-1 [23, 33, 34]. The rat seminal fluid network supports the possibility that high IL-6 and IL-10 levels may circumvent a dependency on eotaxin for recruiting/activating endometrial antigen-presenting cells and eosinophils.

G-CSF was the only cytokine found to be present at significantly higher concentrations in both rat and mouse seminal fluid. Higher G-CSF seminal fluid levels have been reported in fertile compared to infertile men [35], supporting the notion that the maintenance of high G-CSF levels are important in male fertility as well as during the early establishment of pregnancy [15]. Other highly conserved relationships across both body compartments and species was the fact that TNF-alpha consistently featured as the network terminal node. The functional interpretation of this latter observation remains unclear, but has previously been reported in murine lactational networks [22]. The preclusion of feedback loops in the Bayesian network structure means that TNF-alpha’s terminal node status may not reflect a network end point *per se*, but rather that this mediator is under tight regulatory control, although this position has previously been reported in mice [22]. This would be in keeping with studies highlighting TNF-alpha dysregulation as being key to a range of autoimmune disorders, such as rheumatoid arthritis [36]. Its physiological function in rodent seminal plasma remains to be elucidated, and may ultimately be defined through interactions with the endometrium *post coitum*.

Finally, in rat serum, adipose tissue-derived leptin (whose role revolves around energy balance regulation) was present at extremely high levels [37]. Previous studies have described a range in rat circulating leptin concentrations and reported that levels are higher in male rats, where they reflect their adiposity [38, 39]. Although present at comparatively low levels in seminal fluid, the rat seminal Bayesian network suggests that leptin may also participate in regulating seminal cytokine profiles. In this regard, exogenous leptin administration has been shown to reverse the sterility of leptin-deficient obese (*ob/ob)* male mice [40] and improve the motility and viability of human spermatozoa *in vitro* [41]. However, high leptin levels can also have adverse effects on both rat sperm count and morphology [42] and contribute to sperm disorders in obese men [43]. Taken together, these data point to an optimal leptin concentration window required to support normal sperm function which, based on the present findings, may be variably under the influence of IL-4 and IL-12 (p70) in serum and seminal fluid, respectively.

These interpretations have to be considered with three principal caveats. Firstly, as outlined, Bayesian networks preclude the existence of structural feedback loops, such that any given network not based on a time course will present a static snapshot of interrelationships between nodes. Although this offers new insights into the likely causal interrelationships between mediators, these may vary in the *in vivo* setting (e.g. following ejaculation). Moreover, cytokine networks are to some degree dynamic, even in a homeostatic setting, wherein the feedback loops enabling fine tuning of the system are likely not to be captured by the present modelling method. Although beyond the scope of this study, the creation of time series in conjunction with dynamic Bayesian networks may go some way towards clarifying the issue. Secondly, the structure of the networks will inevitably be determined by the array of included mediators. Although this study used the broadest commercially available analytical multiplex panel of cytokines at the time of its inception, it must be acknowledged that the inclusion of additional mediators which interact with those studied herein may result in an altered network structure. Finally, the networks presented are pre-ejaculatory and although they reflect the *status quo* at the level of the male reproductive tract, they cannot predict the dynamic changes in cytokine profile described following maternal tract exposure to seminal plasma [7]. Subsequent validation of the identified mediators is required, either through the use of knock-out mice or exploration of the endometrial response to individual or combinations of mediators.

Another possibility would be to explore gene interactions using Bayesian modelling. From a molecular perspective, cytokines act through their own receptor/s either alone, synergistically, or antagonistically, and activate intracellular pathways (e.g. MAP kinase), which in turn results in the induction/repression of the gene expression of other cytokines (directly or indirectly) and their production at the protein level. This complex scenario is rather simplified in Bayesian networks, which compresses these multiple steps into, effectively, a single edge (i.e. by determining the status of a cytokine node based upon that of its parent/s). As such, the subtlety of aspects such as altered gene expression and mRNA turnover is lost, being amalgamated as conditional probabilities underlying the network structure. However, concentrating on proteins in Bayesian networks is valuable insofar as they go a long way towards capturing some intrinsic features of cytokine interactions, such as synergy and antagonism, which are paramount when evaluating the complex interactions of a specific physiological setting, such as the pre-ejaculatory environment.

## Conclusions

The characterisation of physiological cytokine profiles in seminal fluid using Bayesian models has allowed a more detailed inference of likely inter-mediator causal relationships and highlighted their conservation across species. This method has the advantage of highlighting key regulatory/driver nodes within these inflammatory networks (e.g. MCP-1) which should inform future studies into the validation of these findings in the post-ejaculatory uterine microenvironment.

## Supporting information

S1 Dataset (XLSX)

S1 Fig Prior network used to feed the Bayesian network analysis. The adirectional prior network was constructed using common edges present in both species’ knowledge networks (as directed graphs are never used for seeding). Isolated nodes have as yet no ascribed edges to any other node; these were subsequently learned from the data. Indeed, the final acyclic graphs and underlying conditional probabilities were learned from case-specific data, thereby determining the final network structure with additional/absent edges and directionality.
(TIF)

S1 File Detailed description of Bayesian network formulation. (DOCX)
