# Supplementary material for: A Bayesian view of murine seminal cytokine networks
Source: PLoS One. 2017 Nov 30;12(11):e0188897. doi: 10.1371/journal.pone.0188897 (PMC5708769; doi:10.1371/journal.pone.0188897)
Supplement: S1 File — (DOCX) [file pone.0188897.s003.docx]

**S1 File. Detailed description of Bayesian network formulation**

Bayesian networks are acyclic graphical representations of a network-based hierarchy of interacting variables (as nodes) and their interrelationships (as directed edges representing causal influences). Any given edge has an underlying conditional probability table that defines the probability of the state of a node (relative cytokine concentration, in this setting) given that of its parent(s) immediately upstream – i.e. upstream node(s).

‘Prior’ networks are nuclei around which the remainder of the network can be assembled and their inception has two benefits. On one hand, they account for biologically established interrelationships between variables, the omission of which would fail to incorporate existing knowledge in the field. On the other, they reduce the computational demands allied with the creation of a network. In the present case, prior networks were established based on the cytokines investigated and were learned from a combination of information drawn from literature mining and protein-protein interaction databases using MetaCore and Predictionet, as described in the body manuscript (see S2 Fig for prior network used). Thereafter, these were refined using a machine learning algorithm as part of the MeV package. Prior to performing the Bayesian network analysis, all raw data were normalised to z-scores using Matlab in order to reduce computational expense, a prerequisite for assessing network behaviour. Cytokine profiles were then expressed as relative rather than absolute concentrations and assigned to three mutually exclusive equal width bins (low, intermediate/neutral and high). Subsequently, in the learned networks, each cytokine had an underlying conditional probability table where node colour was determined by its own underlying histogram of distribution across these bins. Since the inherent structure of Bayesian networks cannot incorporate feedback loops (i.e. no node can be either its own ancestor or descendant), any such relationships were eliminated during network assembly.

Network topology and edge-specific conditional probability tables were learned from the data, starting from the initial seed network. Standard non-parametric bootstrapping was applied (100 operations) in order to overcome any possible over-fitting in the analysis. This was achieved by the conventional approach of creating multiple data sets by resampling with replacement to estimate confidence in the network structure. This very robust bootstrapping approach also minimised the number of low confidence edges, even after increasing the confidence stringency to 0.9 (i.e. using features that only occurring in ≥90 % of network iterations). Cytoscape was the used to visualise the networks.

**Definitions**

Node – a mediator, in this case cytokines.

Edge – directional interrelationship between the nodes driven by high confidence levels as determined by stringent bootstrapping.

Parent – a node with no incoming edges but with outgoing edges to one or more nodes. Due to the lack of feedback loops in Bayesian network formulation, this does not indicate that upstream mediators are not present.

Hub – a node which features multiple incoming (>1) input and outgoing edges (≥5). Hub-like nodes have similar features albeit with fewer edges; these have been defined as requiring at >1 input and >1 outgoing edges. These are referred to as ‘hub’ nodes in this manuscript for simplicity since so few fulfilled all the criteria to be defined as true hub nodes.

Terminal node – a node which does not feature any outgoing edges. Due to the lack of feedback loops in Bayesian networks this does not imply an endpoint to a network. However, the multiple influences from other nodes indicates the hierarchy of terminal node regulation.

Orphan – a node which is not connected to other nodes in the network by an edge due to lower levels of confidence following the high stringency iterative bootstrapping process.
